# Integrating terrestrial and aquatic ecosystems to constrain estimates of land-atmosphere carbon exchange

**DOI:** 10.1038/s41467-023-37232-2

**Published:** 2023-03-21

**Authors:** Joan P. Casas-Ruiz, Pascal Bodmer, Kelly Ann Bona, David Butman, Mathilde Couturier, Erik J. S. Emilson, Kerri Finlay, Hélène Genet, Daniel Hayes, Jan Karlsson, David Paré, Changhui Peng, Rob Striegl, Jackie Webb, Xinyuan Wei, Susan E. Ziegler, Paul A. del Giorgio

**Affiliations:** 1 Research Group on Ecology of Inland Waters (GRECO), Institute of Aquatic Ecology, University of Girona, Girona, Spain; 2 Groupe de Recherche Interuniversitaire en Limnologie (GRIL), Département des sciences biologiques, Université du Québec à Montréal, Montréal, QC Canada; 3 Environment and Climate Change Canada, Gatineau, QC Canada; 4 Department of Civil and Environmental Engineering, University of Washington, Seattle, WA USA; 5 Natural Resources Canada, Sault Ste. Marie, Ontario, Canada; 6 University of Regina, Regina, SK Canada; 7 University of Alaska Fairbanks, Fairbanks, AK USA; 8 University of Maine, Orono, ME USA; 9 Umeå Universitet, Umeå, Sweden; 10 Natural Resources Canada, Québec, QC Canada; 11 United States Geological Survey, Boulder, CO USA; 12 Centre for Regional and Rural Futures (CeRRF), Faculty of Science, Engineering and Built Environment, Deakin University, Griffith, NSW Australia; 13 Memorial University of Newfoundland, St. John’s, Newfoundland and Labrador Canada

**Keywords:** Carbon cycle, Environmental sciences

## Abstract

In this Perspective, we put forward an integrative framework to improve estimates of land-atmosphere carbon exchange based on the accumulation of carbon in the landscape as constrained by its lateral export through rivers. The framework uses the watershed as the fundamental spatial unit and integrates all terrestrial and aquatic ecosystems as well as their hydrologic carbon exchanges. Application of the framework should help bridge the existing gap between land and atmosphere-based approaches and offers a platform to increase communication and synergy among the terrestrial, aquatic, and atmospheric research communities that is paramount to advance landscape carbon budget assessments.

## Abstract (web summary)

One of the main sources of uncertainty in carbon budgets is that continental landscapes are made up of a heterogeneous mosaic of ecosystems. Here the authors put forward an integrative framework to improve estimates of land-atmosphere carbon exchange based on the accumulation of carbon in the landscape as constrained by its lateral export through rivers.

## Introduction

Atmospheric carbon dioxide (CO_2_) and methane (CH_4_) concentrations have dramatically increased since pre-industrial times due to anthropogenic emissions^1^, bringing the planet Earth into a climate emergency^2,3^. Developing effective strategies to hold global warming to 1.5 °C and to well below 2 °C above pre-industrial levels—the limits of global average temperature increase established in the UN Paris Agreement^4,5^—has become critically urgent^6^, and hinges upon accurate assessments of the sinks and sources of carbon across the globe. Among the main components of regional and global carbon budgets, the net exchange of carbon between the land and the atmosphere has risen to prominence as being the most uncertain^7^. One of the main sources of uncertainty is that continental landscapes are made up of a heterogeneous mosaic of ecosystems, including forests, wetlands, agricultural lands, inland waters, and other environments that each have their own ecosystem structure and functioning. All of these ecosystems contribute simultaneously to the carbon exchange with the atmosphere, either as sources or sinks. Hence, constraining estimates of land-atmosphere carbon exchange elicits a comprehensive understanding of each of these ecosystems and their associated carbon fluxes, and more importantly, requires a holistic framework that effectively integrates them.

Current methods to quantify land-atmosphere carbon exchange can be generally categorized as either top-down (atmosphere-based) or bottom-up (land-based)^8^. Top-down methods estimate the land-atmosphere exchange of carbon by utilizing atmospheric concentration measurements and atmospheric transport models. This approach measures “what the atmosphere sees”, in the sense that it captures the true entirety of carbon exchanges between the landscape and the atmosphere as one integrated flux. Bottom-up methods, on the other hand, combine estimates of net ecosystem carbon exchange (NEE; Box 1) for individual ecosystem types (e.g., forests, wetlands, croplands, lakes) to reconstruct the carbon exchange between the atmosphere and the landscape as a whole. Therefore, the accuracy of bottom-up methods is highly sensitive to how well different landscape ecosystems and processes are represented. Individual NEE estimates are commonly derived from carbon inventories, remote sensing, process-based models, or direct measurements using eddy covariance towers or flux chambers^8^. While top-down methods integrate all fluxes involved in the land-atmosphere exchange of carbon, they provide limited attribution or understanding of the ecosystems and processes involved. Conversely, bottom-up approaches often overlook some processes and ecosystems but provide mechanistic insights, and hence allow assessment of potential management strategies to reduce emissions or promote carbon sinks on the landscape.

Despite steady progress in estimating land-atmosphere carbon exchange, there is still a significant degree of discrepancy between top-down and bottom-up methods^1,9^. There are several underlying reasons for the deviations between these two approaches as, for example, the fluxes derived from land use changes and wildfires^10^. These fluxes are well captured by atmospheric inversion models (AIMs) but are not well represented in process-based, biospheric models. Another major issue, and arguably the most challenging one to address in bottom-up methods, is the incomplete representation of the role that water plays in the landscape^11^. Streams, rivers, and lakes occupy only a small fraction of the Earth’s non-glaciated continental surface (2.7%; ref. ^12^), however they emit a disproportionate amount of greenhouse gases to the atmosphere^13^. It is estimated that inland waters emit globally around 3.9 Pg C yr^−1 ^^14^, which is in the same order of magnitude as current estimates of carbon uptake by the world’s forests (2.4–7.6 Pg C yr^−1^; refs. ^15,16^) or the emissions derived from wildfires globally (2.2 Pg C yr^−1^; ref. ^17^). An additional unknown is the extent and distribution of artificial water bodies created for human purposes (e.g., artificial lakes, farm dams, agricultural channels, urban aquatic systems) and how these contribute to the global carbon budget (but see ref. ^18^). The role of inland waters as significant sources of carbon to the atmosphere is today well recognized by the scientific community, and aquatic carbon emissions are starting to be considered in national, regional, and global assessments of landscape-atmosphere exchange (e.g., SOCCR2; IPCC; RECCAP-2).

Far less represented is the role that water plays in moving carbon laterally across the landscape. A significant fraction of the carbon fixed and processed in terrestrial ecosystems is mobilized via surface runoff and groundwater flows into inland waters, from where it can be not only emitted back to the atmosphere but also stored in aquatic sediments or transported downstream. Therefore, changes in terrestrial carbon stocks do not result exclusively from ecosystem metabolism and exchange with the atmosphere but also from the lateral transfer of carbon to inland waters via hydrologic transport^19–21^. This has important implications for bottom-up assessments of land-atmosphere carbon exchange. On the one hand, terrestrial carbon inventories and models track the total carbon stock loss in forests and wetlands but generally do not distinguish between direct losses to the atmosphere and losses to inland waters (e.g., CBM-CFS3; ref. ^22^). Hence, some of the carbon that inventories and models assume to be emitted from terrestrial ecosystems has been relocated and is in reality emitted through inland waters. This implies that the indiscriminate addition of aquatic carbon emissions to those calculated or modeled for terrestrial systems may lead to a double carbon accounting issue. On the other hand, overlooking the lateral transfer of carbon from terrestrial to inland waters can highly bias terrestrial NEE estimates^23–25^. For example, the hydrologic transfer of organic carbon from forests and wetlands to streams represents a direct removal of terrestrial net primary production (NPP). Thus, ignoring this lateral transfer will lead to an underestimation of terrestrial net ecosystem production (NEP; often assumed equivalent to NEE) in process-based models and inventory-based methods. Recent research of the Amazon basin has indeed shown that ignoring hydrologic carbon transfers leads to an underestimation of the basin NEP by 8.6%^23^. In all cases, in addition to its quantification, understanding the fate of the carbon transferred from terrestrial to aquatic systems is paramount to effectively incorporate this lateral flux in landscape carbon budget assessments (Box 2).

Correctly accounting for the hydrological connectivity between terrestrial and aquatic ecosystems is critical to improve estimates of land-atmosphere exchange derived from inventories and process-based methods. Nevertheless, whereas much research has been devoted to this topic over the last decade (e.g., refs. ^26–32^), our understanding of the hydrological export of terrestrial organic and inorganic carbon to inland waters is still limited and highly uncertain, especially at regional and continental scales (but see refs. ^23,33^). This is well reflected, for example, in the last assessment of the North American Carbon Budget (SOCCR2; ref. ^34^). In that synthesis effort, amongst all evaluated fluxes, the hydrological export of terrestrial carbon was assigned the lowest level of confidence, with high impact on the reliability of land-atmosphere carbon exchange estimates. This very low confidence is unlikely to improve in the next few years due to the multitude of methodological challenges associated with the characterization of lateral hydrologic carbon transfers, particularly those associated with groundwaters. Hence, there is a need for complementary bottom-up approaches that integrate all the different ecosystems in the landscape but that do not rely on specific estimates of the lateral transfer of carbon from individual ecosystems.

A basic and effective way to indirectly capture all the ecosystems in the landscape as well as their hydrologic carbon exchanges is through a mass balance method for the landscape as a whole. Therefore, we argue that whole-landscape carbon mass balances should offer a complementary, independent approach to estimate land-atmosphere carbon exchange as well as a unique opportunity to constrain inventory-methods and improve process-based models. A recent successful example can be found in the work by Ciais et al. ^35^, in which continental mass balances of carbon were combined to derive a new bottom-up estimate of the global exchange of carbon between the land and the atmosphere. This new estimate was in turn used to constrain estimates of the global soil heterotrophic respiration, one of the largest and perhaps the most uncertain components of the continental carbon cycle^35^. While there is a great potential in the use of mass balances for landscape carbon accounting, their application is far from straightforward, and this may explain why the approach has not been widely applied. The number, type, and relative influence of carbon fluxes into and out of a landscape unit differ across scales and biomes, and so does the heterogeneity and composition of the landscape mosaic. Moreover, whether the spatial unit of the mass balance is defined based on political, hydrological, or arbitrary boundaries further determines the structure and complexity of the mass balance equation. In this regard, given the challenges associated with hydrologic carbon fluxes across ecosystems, it is relevant to choose a spatial unit that is hydrologically meaningful, and which allows for simplifications of the carbon mass balance across scales. Finally, successful application of whole-landscape carbon mass balances for landscape carbon accounting not only requires a proper understanding of the landscape composition and connectivity, but also increased coordination and synthesis between the different terrestrial and aquatic research disciplines.

Here we put forward the Net Watershed Exchange (NWE) concept, a framework to facilitate the application of whole-landscape mass balances to estimate the contemporary land-atmosphere exchange of carbon. Partial formulations of this basic concept have been deployed in the past^35–40^, yet this literature still lacks a robust and unifying conceptual backbone that allows application across scales and landscape types. Our goal here is to formalize this rather fragmented field into a coherent framework that can be effectively carried forward and stimulate new research venues. The proposed framework explicitly integrates aquatic and terrestrial ecosystems and uses the watershed as the central spatial unit of the mass balance, which simplifies the representation of water and carbon lateral flows across the landscape and overcomes the main challenges associated to the hydrologic exchange of carbon among ecosystems (i.e., potential for double carbon accounting and biased terrestrial NEE and NEP estimates; see above). With this framework, we aim to move away from the traditional compartmentalized view of the continental carbon cycle, where each ecosystem is considered in isolation, and move toward a more holistic perspective of the landscape functioning. In the next sections we build, step-by-step, the mass balance underlying the framework, elaborate on how it overcomes some of the current challenges of landscape carbon accounting, discuss its advantages and limitations, and provide a roadmap for its application and future improvement.

Box 1 Glossary ***Eddy covariance towers:*** Sensor towers that measure the net exchange of carbon (CO_2_ and/or CH_4_) between the land and the atmosphere at the ecosystem scale. These provide direct measurements of NEE with footprints on the order of 1 or more square kilometers.

***Double carbon accounting:*** Accounting twice for the same carbon fluxes across the landscape, commonly caused by insufficient communication and synthesis between different research disciplines.

***Net ecosystem productivity (NEP):*** Balance between gross primary productivity and respiration (autotrophic plus heterotrophic) of an ecosystem. Positive values indicate net uptake of carbon by the ecosystem.

***Net ecosystem exchange (NEE):*** Exchange of CO_2_ between an ecosystem and the atmosphere. In addition to NEP, which is a biological process, NEE also involves abiotic pathways of CO_2_ release and uptake such as wildfires and mineral weathering. NEE was defined by atmospheric scientists as a carbon input to the atmosphere, thus by convention positive values indicate emission to the atmosphere (opposite from NEP). In some studies, the term NEE has been reused for the vertical carbon exchange of whole regions and continents, with all ecosystem types included. To avoid potential confusion, in this manuscript we stick to the original definition of NEE as an individual ecosystem flux and clearly specify if otherwise (e.g., Watershed NEE, Continental NEE).

***Terrestrial biosphere models (TBMs):*** Process-based models that explicitly couple biogeography, biogeochemistry, biophysics, and vegetation dynamics to estimate stocks and fluxes of matter in terrestrial ecosystems. TBMs generally operate at the continental and global scales.

***Net watershed exchange (NWE):*** NWE is operationally defined as the net exchange of carbon between the whole surface of a watershed and the overlying atmosphere calculated using a mass balance approach (Eq. 3). NWE is defined as positive when there is a net flux from the atmosphere to the land (i.e., a net input to the watershed).

***Net ecosystem carbon balance (NECB):*** Change of carbon stock per unit time over an ecosystem, calculated through a carbon mass balance where all fluxes are represented in the same spatial and temporal integrated units. Both vertical and lateral fluxes are included. Positive NECB values indicate carbon accumulation in the ecosystem, whereas negative values represent a carbon loss.

***Atmospheric inversion models (AIMs):*** Top-down method to estimate the land-to-atmosphere net carbon fluxes by utilizing atmospheric CO_2_ and/or CH_4_ concentration measurements and atmospheric transport models constrained by a priori knowledge of sources and sinks.

***Sediment focusing:*** Sediments being unevenly deposited across a lake bottom due to post-depositional horizontal transport and lake basin heterogeneity.

Box 2 The fate of terrestrial carbon in inland waters A share of the atmospheric carbon taken up by terrestrial ecosystems is mobilized through hydrologic flows into inland waters. Once in inland waters, whether this carbon is emitted back to the atmosphere or buried in aquatic sediments has contrasting implications that need to be considered when integrating terrestrial and aquatic carbon fluxes. Firstly, a significant fraction of aquatic emissions is simply a relocation of recent terrestrial respiration that would otherwise be emitted to the atmosphere directly from terrestrial systems^73,179^. These are the carbon emissions that are often double counted in landscape carbon budget assessments, particularly when terrestrial models and inventories do not distinguish between direct respiration losses to the atmosphere and losses to inland waters. Secondly, another fraction of stream, river, and lake carbon emissions derives from the mineralization of terrestrial organic carbon within aquatic systems^180^, which in this case represents a conversion from terrestrial NPP to aquatic heterotrophic respiration. Thirdly, some of the carbon that did not accumulate in the terrestrial domain may be transported short to long distances through streams and rivers and eventually accumulate in lake sediments^143^, where dark, cold, and oxygen-limited conditions slow down or prevent its mineralization^181^. The aquatic emissions resulting from the first and second cases reflect the role of inland waters as vents and reactors of recently sequestered terrestrial carbon, contributing to the short-range, rapid cycling of carbon between the landscape and the atmosphere^46^. Burial in lakes, in contrast, results in the transfer of terrestrial carbon to a long-term storage compartment, removing it from the contemporary land-atmosphere carbon exchange. Therefore, besides transferring carbon from land to ocean, inland waters play a dual role within the landscape as short-term sources and long-term sinks of terrestrially derived carbon, in many ways as an extension of soil processes. Understanding the diverse fates of the lateral carbon transfer from terrestrial to aquatic systems is necessary to fully incorporate this dual role of inland waters into landscape carbon budget assessments as well as to avoid potential issues of double carbon accounting.

## The Net Watershed Exchange framework

### A simple mass balance approach

From a mass balance point of view, the net accumulation of carbon through time in a given landscape unit should equal the balance between its carbon inputs and outputs. Thus, if we separate vertical and horizontal fluxes, the net carbon accumulation in the landscape should be equivalent to the net exchange with the atmosphere plus the net balance between lateral carbon inputs and outputs. The net carbon accumulation in the landscape can be understood as the combined accumulation in all the different ecosystems that constitute the landscape, for example, through the accrual of carbon in soils and wetlands, the growth of above and belowground biomass in forests or the storage of carbon in the bottom of lakes and reservoirs. Lateral fluxes, in turn, basically include the hydrological carbon fluxes into and out of the landscape unit as well as anthropogenic lateral fluxes such as the trade of wood, fiber, and food products.

In the absence of anthropogenic lateral fluxes, we can express a short-term landscape carbon budget as:

1 $$\triangle C={LandAtm}+{H}_{{in}}-{H}_{{out}}$$

where Δ*C* is the net accumulation (or loss) of carbon through time in the whole landscape unit, *LandAtm* is the land-atmosphere carbon exchange (defined here as positive when there is a net flux from the atmosphere to the land), and *H*_*in*_ and *H*_*out*_ represent the lateral hydrologic inputs and outputs of carbon. The formulation in Eq. 1 is, in essence, a simplified landscape-scale version of a net ecosystem carbon balance (NECB), sometimes also referred to as net biome production (NBP) when applied at large temporal and spatial scales. These integrated carbon cycling terms were introduced by Chapin et al.^20^ as a framework to assess ecosystem carbon cycling in a more holistic way, reconciling carbon fluxes into and out of an ecosystem with the rates of carbon accumulation. NECBs have been applied at the ecosystem level to assess carbon cycling in a multitude of environments^41^, including forests^42^, peatlands^43^, agricultural fields^38^, coastal wetlands^44^, and lakes^45^. Nonetheless, it is only recently that the NECB concept has been extended to the landscape level (net landscape carbon balance; refs. ^37,39^). One of the main reasons why NECBs have seldom been applied at the landscape level is that, as mentioned above for the case of individual ecosystems, hydrological carbon fluxes into and out of a landscape unit (*H*_*in*_ and *H*_*out*_ in Eq. 1) are rather challenging to pinpoint, quantify, and trace^46^. This is particularly true for landscape units that are defined based on political boundaries or arbitrary spatial units such as grid cells, because water does not follow nor stop at human-defined borders. This challenge becomes much smaller, however, if the mass balance is performed in a hydrologically defined landscape, for example in a watershed.

**Fig. 1:**
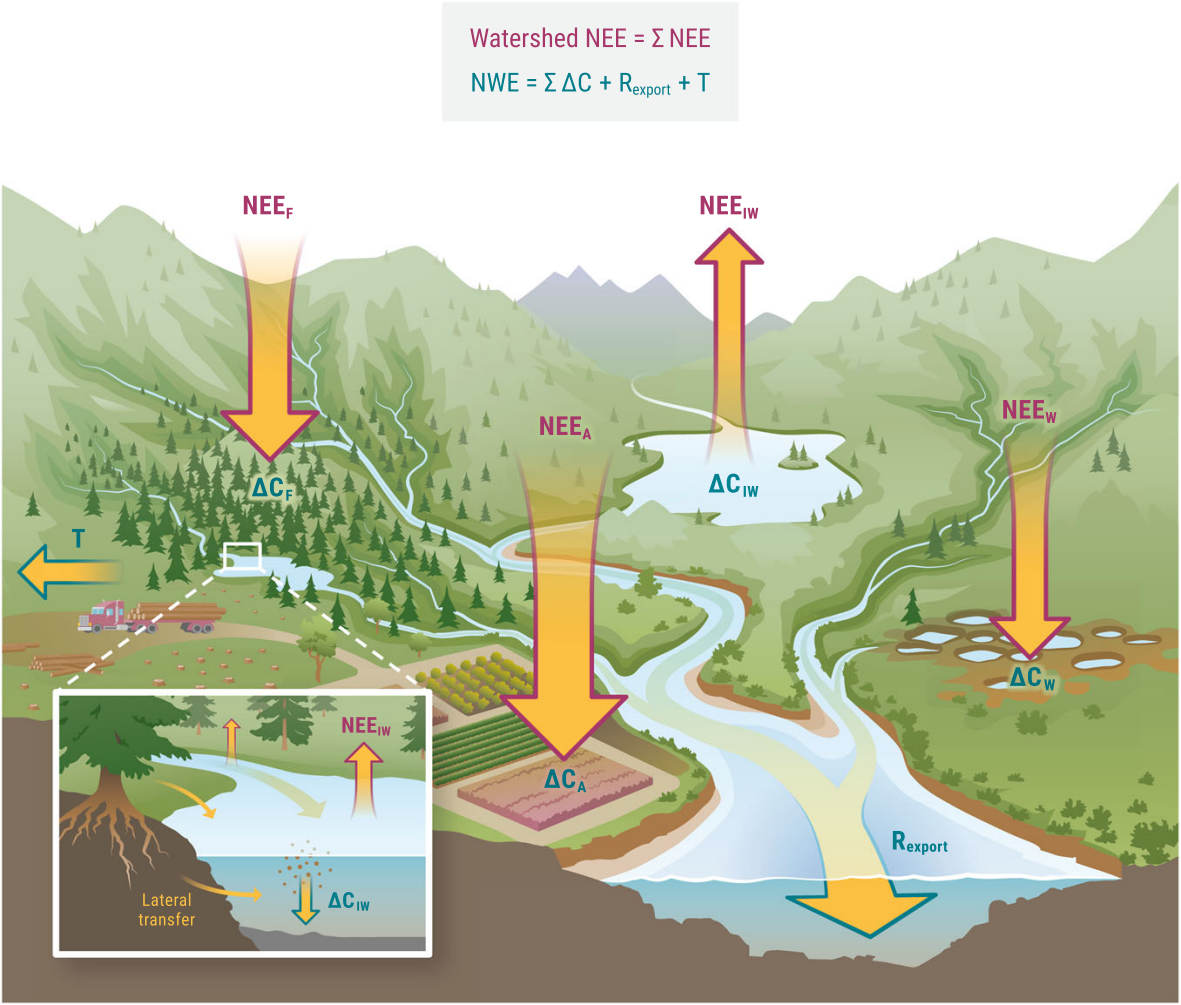
Carbon fluxes across the atmosphere-land-water continuum of a watershed. Conceptual diagram of the vertical and lateral flows of biospheric carbon across a watershed, depicting the most common source/sink role of different terrestrial and aquatic ecosystems. Highlighted in color are the elements involved in the calculation of land-atmosphere carbon exchange either as Watershed NEE (*∑NEE*; in purple) or NWE (Eq. 3; in dark turquoise). Carbon is withdrawn from the atmosphere through the photosynthesis of forests, wetlands, agricultural lands, and other terrestrial ecosystems. Yet not all the carbon accumulates or returns to the atmosphere in situ. A significant fraction of terrestrial productivity and respiratory CO_2_ is hydrologically transferred to inland waters, from where it can be emitted back to the atmosphere, stored in aquatic sediments, or routed downstream towards the watershed outlet (inset). Since inland waters receive external carbon inputs, they are generally net sources of carbon to the atmosphere while at the same time they act as long-term sinks of terrestrially derived carbon. The riverine carbon export at the watershed outlet (*R*_*Export*_) integrates the transfer, routing, and cumulative processing of terrestrial carbon through the entire aquatic network, and it can be seen as the imbalance between the net watershed carbon exchange with the atmosphere and the total watershed carbon accumulation. In managed landscapes, the harvesting and trading (*T*) of wood and crops involve additional lateral carbon fluxes that need to be accounted for in watershed carbon budget assessments. Non-biospheric carbon fluxes derived from volcanism, chemical weathering, and petrogenic organic carbon oxidation may also significantly contribute to contemporary land-atmosphere carbon exchange. NEE Net Ecosystem Exchange, NWE Net Watershed Exchange, ∆*C* ecosystem carbon accumulation (or loss), W wetlands, IW inland waters, A agroecosystems, F forests. Figure by Visualizing Science.

A watershed (or drainage basin or catchment) is a landscape unit that reflects the natural routing of water. It is defined as an area of land where all the water that falls in it (and not returned to the atmosphere via evapotranspiration) is routed from terrestrial through aquatic systems toward a single exit point, namely the watershed outlet. Using a watershed as the landscape unit of a mass balance has two advantages. First, besides some exceptions (see “Challenges and opportunities” in the Supplementary Information), hydrological exchanges between adjacent watersheds are generally very minor relative to other terms of the watershed carbon balance. Hence, hydrological inputs of carbon into watersheds (*H*_*in*_ in Eq. 1) can be assumed to be negligible. Second, virtually all of the carbon that is hydrologically exported out of a watershed (*H*_*out*_ in Eq. 1) passes through the outlet, usually the mouth of a stream or river. Thus, the accumulation of carbon in a watershed ultimately results from the balance between the net carbon input from the atmosphere and the carbon export through the riverine outlet (Fig. 1). We can therefore rearrange the mass balance equation and estimate the contemporary carbon exchange between the watershed and the atmosphere:

2 $${NWE}=\mathop{\sum}\limits_{i=1}^{n}{\triangle C}_{i}+{R}_{{Export}}$$

where *NWE* is the net vertical exchange of carbon between the whole surface of a watershed and the atmosphere directly above it, ${\sum }_{i=1}^{n}{\triangle C}_{i}$ is the combined net accumulation (or loss) of carbon in the different terrestrial and aquatic ecosystems within the watershed, and *R*_*Export*_ is the riverine export of carbon at the watershed outlet. Units are carbon mass per year (if possible, the average of at least a decade; see next section) and are the same for all equation terms. The index *n* represents the number of different terrestrial and aquatic ecosystem types (e.g., forests, wetlands, lakes, grasslands, agroecosystems) in the landscape, and will vary depending on the landscape configuration of each study case. In Eq. 2, NWE is positive when there is a net flux from the atmosphere to the watershed (Box 1), ∆*C* for each ecosystem type is positive when carbon storage increases (i.e., stock change > 0), and *R*_*Export*_ is always positive. In some landscapes, the watershed outlet may not be an actual river but agricultural or coastal canals. Note also that in the case of endorheic watersheds there is no watershed outlet, thus NWE should equal the carbon accumulation in the different terrestrial and aquatic ecosystems within the watershed $({NWE}={\sum }_{i=1}^{n}{\triangle C}_{i})$.

Besides hydrologic carbon fluxes, there are additional lateral fluxes of carbon that need to be accounted for in a landscape mass balance. The trade of wood, fiber, and food products represents a major anthropogenic lateral flux of carbon across scales^47^ and is an important component of regional and continental carbon budgets^48^. These lateral fluxes are unrelated to the direct exchange of carbon between the watershed and the atmosphere; however, they do represent a carbon gain or loss that is reflected in carbon stock changes and therefore in the net accumulation or loss of carbon within the watershed. Hence, to estimate NWE using a mass balance, lateral transfers of product carbon must be brought back into the equation as follows:

3 $${NWE}=\mathop{\sum}\limits_{i=1}^{n}{\triangle C}_{i}\,+{R}_{{Export}}+T$$

where *T* represents the net trade of product carbon (defined as exports minus imports). Note that ${\sum }_{i=1}^{n}{\triangle C}_{i}$ should now also include the carbon-stock change in wood and crop products in the watershed, including products decaying in landfills. Other lateral carbon fluxes into and out of the watershed such as wind transport^49^ or insect outbreaks and migration^50^ could be also incorporated into the calculation as additional terms if relevant.

The mass balance underlying Eq. 3 accounts not only for biotic but also abiotic pathways of carbon uptake and release, as long as these translate into changes in the watershed carbon stocks or riverine carbon export. For example, wildfire carbon emissions are captured by the accumulation (or loss) and *R*_*Export*_ terms in Eq. 3, because they translate into a drastic reduction of forest carbon stocks as well as into riverine export of pyrogenic “black” carbon^51^. Equation 3 also accounts for the uptake of atmospheric CO_2_ by silicate and carbonate rock weathering, a geological carbon flux that impacts present day atmospheric CO_2_. Geological pathways of CO_2_ release to the atmosphere such as petrogenic (rock-derived) organic carbon oxidation or sulfide oxidation are not captured by Eq. 3 as is, but they can be incorporated into the watershed mass balance as additional terms (see next section).

Except for the sign convention, NWE is conceptually equivalent to what could be called watershed NEE, that is, the sum of the different terrestrial and aquatic NEEs within the watershed (Fig. 1). The difference between these two concepts is purely operational, as it rests solely on the mathematical approach used. This implies that quantifying land-atmosphere carbon exchange as either Watershed NEE or NWE requires a completely different set of data. Watershed NEE relies on estimates of the vertical carbon exchange of individual ecosystems (NEE), whereas NWE requires estimates of ecosystem carbon accumulation in addition to riverine export. Both approaches have their own set of advantages, limitations, and methodological challenges (see sections below), but are complementary to each other. The NWE framework provides an alternative method to quantify land-atmosphere carbon exchange that can be applied across scales, but more importantly, it provides an independent constraint to improve traditional bottom-up approaches.

### On the spatial and temporal dimensions of the NWE framework

The NWE framework applies to all spatial scales, from very small watersheds of a few hectares to watersheds of thousands of square kilometers, because the size of the watershed does not affect the principles underlying its base equation (Eq. 2). In fact, it is not strictly necessary to adhere to the watershed as the only spatial unit. Other large-scale units can also be used as long as they are defined based on hydrology, for example the Hydrologic Units (and their sub-divisions) in USA, the global COSCATs segmentation scheme^52^, or even a whole continent (see ref. ^35^ for a pioneer application of a carbon mass balance to estimate net continental carbon exchange). The only particularity in such cases is that there may not be a single outflow but rather several rivers (or canals) draining the unit, and thus hydrological carbon exports should be quantified as the combined export of these rivers.

As for the temporal dimension of the mass balance, longer time scales that capture temporal variability in carbon fluxes are recommended. First, an important share of the land-atmosphere carbon exchange can be episodic, occurring in very narrow time frames through disturbances and extreme climatic events. For example, in just a few days or weeks, wildfires may release to the atmosphere vast amounts of carbon that took decades or even centuries for forests to accumulate^17^. Thus, computing the mass balance at longer time scales increases the chance of capturing natural and anthropogenic disturbances that are infrequent but may nevertheless strongly impact the land-atmosphere carbon exchange of a landscape. Second, the accuracy of carbon stock change estimates for forests, wetlands, and lakes increases when using longer time frames of integration^53,54^. It is often challenging to meaningfully quantify or even detect seasonal and yearly changes in these stocks. Last, there can be a significant time lag between NWE and *R*_*Export*_. A drop of water may need on average anywhere from weeks to decades to travel from soils through drainage networks to the river mouth^55^, depending on watershed geomorphology and the presence of large lakes with extended water residence times. As a result, changes in NWE and *ΔC* derived from natural oscillations such as interannual variability or El Niño-Southern Oscillation do not immediately translate into changes in *R*_*Export*_. Therefore, even though steady state conditions are assumed (i.e., all fluxes are constant through time), we reduce the risk of bias by increasing the timescale of the mass balance. According to Downing and Striegl^56^, 80% of the groundwater carbon flux to inland waters occurs from the upper 40 m of groundwater, where the average water turnover time is 6–14 years. For these reasons, using data that cover seasonal and interannual variability across decades or longer time scales will yield more robust estimates of NWE. We therefore recommend making the mass balance calculations (Eq. 3) using the average of at least ten years if possible. Using a decadal time scale has the additional advantage in that it renders NWE estimates comparable with international assessments on climate change and carbon cycling such as the IPCC’s Physical Science Basis report^1^, the Global Carbon Budget^7^, or the SOCCR2^34^, which are currently budgeting 10-year periods.

### Inputs of old carbon to inland waters

Most of the carbon flowing through inland waters is modern and ultimately originates from recent watershed NPP. Some rivers, however, receive significant amounts of ancient carbon from the lithosphere that are disconnected from the contemporary atmospheric CO_2_ uptake, and which require particular attention. Potential sources of lithospheric carbon to inland waters include, (i) the remobilization of petrogenic (rock-derived) organic carbon^57^, which can eventually be oxidized and emitted to the atmosphere, and (ii) the dissolution of carbonate minerals, either through reaction with soil-respiratory CO_2_ or with sulfuric acid derived from sulfide mineral oxidation^58^. The contemporary aquatic emission, storage, and export of this ancient carbon are captured by the different terms in Eq. 3; however, its input from the lithosphere is not, thus leaving the short-term carbon mass balance unclosed. This implies that, given that Eq. 3 assumes all carbon inputs to the watershed come from the atmosphere, inputs of ancient carbon from the lithosphere would be mistakenly accounted as contemporary watershed NPP, potentially leading to an overestimation of NWE. To effectively account for present-day sources of lithospheric carbon to inland waters, we recommend the inclusion of additional input terms to Eq. 3 (see Supplementary Information for guidelines). The significance of lithogenic carbon sources to inland waters might vary greatly across regions and biomes depending on watershed geomorphology. Inputs of petrogenic organic carbon to inland waters are tightly linked to the erosion of sedimentary rocks, and should receive special attention in mountain watersheds underlain by sandstones and shales^59^. Inputs of ancient inorganic carbon from weathered carbonates will be particularly relevant in watersheds dominated by carbonate lithologies.

Human disturbance and climate change may additionally induce the remobilization of aged (hundreds to thousands of years old) organic carbon from soils into aquatic networks. This is especially true in watersheds with intense deforestation and land degradation^60^, as well as in northern and alpine regions affected by permafrost thaw^61,62^. Inputs of aged soil organic carbon would have the same implications for NWE estimates as lithospheric carbon sources and should ideally be accounted for in the same way. Nevertheless, quantifying the remobilization of aged soil carbon at the watershed scale remains a major methodological challenge. Hence, new advancements are required before aged soil organic carbon sources can be systematically accounted for within the NWE framework.

## Advantages and limitations of the NWE framework

A key advantage of the NWE framework compared to current bottom-up methods is that it does not rely on estimates of the lateral hydrologic transfer of carbon among ecosystems, because these are implicit in the whole-watershed carbon mass balance (see Supplementary Note for a numerical demonstration). Hence, besides bypassing the substantial challenge of quantifying the lateral transfer of terrestrial carbon to aquatic systems, the NWE approach avoids the risk of double accounting the same carbon fluxes across the landscape.

Another advantage of the framework is that it is based on carbon accumulation, which in many terrestrial ecosystems is often less uncertain and more straightforward to measure than NEE. In forests, for example, repeated carbon inventories of live and dead biomass, litter, soil carbon, and wood and crop products^8^ provide, despite their intrinsic technical challenges, a simple method to estimate carbon accumulation without the necessity of running complex process-based models or deploying expensive eddy covariance tower networks in the field. In this regard, the NWE framework opens new opportunities for landscape carbon accounting across the globe, including in funding-constrained developing countries^63^. Data on carbon accumulation in wetlands and lakes are less common, partly because most research efforts in the last decades have primarily focused on NEE or have been limited by methodological challenges^64^. In this regard, a critical line of future research is to advance the understanding and quantification of carbon accumulation in the different wetland and aquatic components of the landscape (see section “The way forward” for a detailed discussion).

An additional opportunity provided by the NWE framework is in addressing other questions concerning landscape carbon accounting. In particular, if the carbon exchange between watershed and atmosphere is measured through independent methods such as eddy covariance towers or AIMs, an arrangement of Eq. 3 would allow the inference of other components of the mass balance that may remain unresolved (e.g., carbon accumulation in wetlands).

The NWE framework has limitations that also need to be considered. First, it is based on a mass balance of total carbon and does not track the conversion between the different forms of carbon, for example from CO_2_ to organic carbon through photosynthesis or from organic carbon to CH_4_ via methanogenesis. Thus, the framework per se cannot differentiate how much of the carbon exchange with the atmosphere occurs in the form of CO_2_, CH_4_, or other carbon forms such as organic volatiles. This is a drawback of the approach, because despite the fact that most natural and anthropogenic carbon emissions occur as CO_2_, CH_4_ has a much greater warming potential—34 times higher than CO_2_ in a 100-year horizon^65^—and is therefore fundamental for future climate predictions^66^. That said, if the exchange of CO_2_ and organic volatiles between the landscape and the atmosphere are well constrained, these data could be used in combination with Eq. 3 to derive the land-atmosphere exchange of CH_4_ by difference.

Second, the NWE framework provides estimates of whole-watershed carbon exchange with the atmosphere, but it cannot parse out the contribution of the different ecosystems within a watershed to the total carbon exchange (i.e., their individual NEE). Thus, it does not provide information on where in the watershed carbon is withdrawn from or released to the atmosphere. Yet, the framework does inform where and how much carbon accumulates, which is in fact more important for developing climate mitigation strategies that aim to remove atmospheric CO_2_ by enhancing natural carbon sinks^67^. For such nature-based strategies to be effective, it is paramount that the CO_2_ is stored in the landscape and does not return to the atmosphere, at least on a short-term basis. For example, mitigation activities such as deforestation avoidance, reforestation, and afforestation count on a positive effect of increased atmospheric CO_2_ concentrations on the photosynthetic uptake of CO_2_ by forests^68–70^, a process referred to as the fertilization effect. However, recent ecosystem-scale experiments in mature forests have demonstrated that this positive effect on carbon uptake does not necessarily translate into increased tree growth and carbon sequestration^71,72^. Instead, most of the extra carbon taken up can be shortly released back to the atmosphere through root and soil respiration, thereby reducing the predicted efficacy of these climate mitigation activities^72^. Similarly, a potential increase in atmospheric carbon uptake by forests and wetlands would be in vain if this extra carbon is not stored but rapidly transferred to aquatic systems and emitted back to the atmosphere^73^. Hence, nature-based, climate mitigation strategies would greatly benefit from optimizing carbon accumulation rather than uptake. In this regard, the NWE framework offers a tool to understand where and how much atmospheric carbon is actually sequestered within the landscape, which can be used to support decision making.

A third limitation of the framework is that by definition watersheds are scale invariant, but land ownership is not. Implementation of strategies to account for and manage carbon will no doubt be defined across many different environmental and political boundaries. The NWE framework is a method to account for all carbon but calls inevitably for a more unified set of monitoring and verification procedures across political boundaries.

## A sideways solution to a bottom-up vs. top-down problem

For scientific study and policy analysis purposes, carbon budget assessments are often performed at regional scales and summarized over annual to decadal time periods^8,74^. Inventory-based methods^75,76^ are often favored for broad-scale carbon budget assessments based on their use of a large number of measurements, ability to track the total carbon stock, and comparability among estimates. Inventories generally track changes in carbon pool sizes using data collected for the major land-based sectors, such as managed forests and agriculture. When aggregating across these sectors for a regional scale estimate of the land carbon sink, a major source of uncertainty stems from those components of the budget that are potentially important but are not measured or otherwise estimated by the inventories. These components include fluxes from unmanaged and non-inventoried lands (wetlands, rangelands), potentially important mechanisms not captured (woody encroachment on non-forest landscapes), other potential carbon storage pools (lakes and reservoirs), and lateral hydrologic fluxes (carbon export from soils and wetlands through rivers to the ocean). Terrestrial biosphere models (TBMs), meanwhile, can operate globally and simulate both net biological carbon uptake from the atmosphere (NEP) and total ecosystem carbon change (*ΔC*), but otherwise differ widely in their structural designs and processes included^77^.

Attempts to reconcile the carbon budget estimates among different approaches have primarily focused on synthesizing and comparing estimates of land-atmosphere carbon exchange from top‐down versus bottom‐up methodologies^11^. Previous comparisons with AIMs (top-down) have suggested a much stronger land CO_2_ sink than can be accounted for in bottom-up inventories and TBMs at either regional^78–80^ or continental scales^9,81^. Comparisons in this direction have suggested that a large contribution of the non-inventoried fluxes would need to be added to the inventory-based estimates to approach the magnitude of the continental-scale carbon sink indicated by the AIM and TBM model ensembles^82^. Indeed, these regional- and continental-scale carbon budget synthesis efforts have demonstrated the importance of carefully and consistently including the lateral transfers of carbon to bridge the land-atmosphere exchange estimates^8,35,83^. In particular, whereas biomass stocks and harvested products often receive more attention in greenhouse gas accounting^84^, the lateral transfer of terrestrial carbon to aquatic systems is critical to account for where they are otherwise assumed to be lost to the atmosphere or stored in the ecosystem^85,86^. Given the large uncertainty in TBMs and the potential for missing components in inventories, including lateral aquatic carbon transfers into bottom-up methods and comparing these with top-down estimates is essential to provide an overall constraint on the land carbon sink.

In concept, AIMs should not be missing any major carbon budget components since they “see” all land-atmosphere exchange as one integrated flux, and thus represent an overall top-down constraint on the regional carbon budget. Similarly, the riverine export of carbon at the watershed outlet integrates the routing and processing of terrestrial carbon through the entire aquatic network. Hence, riverine export (*R*_*Export*_ in Eq. 3) integrates across ecosystem types, and it “sees” the net result of all aquatic lateral transfers of carbon out of terrestrial ecosystems within the watershed (Supplementary Note). Thus, just as AIMs provide a top-down estimate of carbon exchange over a region of interest, the measurement of the amount of carbon exported through watershed river outlets provides a key “sideways” constraint on the broad-scale budget when summed over all watersheds within the region.

We can look at efforts to reconcile the continental CO_2_ sink of North America as an example of how riverine carbon export can be used as a sideways constraint for inventory-based methods. In a progression of studies over time, top-down estimates of the mean land sink for North America have decreased from 1700 ± 500 Tg C per year^87^ to 890 ± 409 Tg C yr^−1^ in the Regional Carbon Cycle Assessment and Processes project^9^, and to 699 ± 82 Tg C yr^−1^ in the updated SOCCR2^88^. Meanwhile, the total carbon exported by North American rivers to the coastal ocean is estimated at 106 ± 30 Tg C per year^89^. Hence, according to the NWE framework (Eq. 3), 593 ± 87 Tg C yr^−1^ should be stored annually in the different carbon storage pools within the North American continent. This number can subsequently be used as a reference for the evaluation and improvement of inventory-based assessments of land-atmosphere carbon exchange. For example, in the updated SOCCR2, adding up the terrestrial sector inventories, wood products pools, and carbon burial in lakes, reservoirs, tidal wetlands, and estuaries accounts for 496 Tg C yr^−1^ of storage^83^. The general convergence with the independent top-down estimate of carbon accumulation as constrained by riverine export (593 ± 87 Tg C yr^−1^) gives us confidence that we are at least not missing any major components in the SOCCR2 inventory-based accounting, yet the differences that persist should motivate and guide efforts to improve current estimates of carbon storage across ecosystems.

The NWE framework can also help constrain TBMs when used over hydrologically defined landscapes. Most TBMs will report the mass balance of landscape carbon (i.e., ${\sum }_{i=1}^{n}{\triangle C}_{i}$ in Eq. 3), but modelers should be encouraged to simulate land-atmosphere exchange and *R*_*Export*_ as well (see e.g., ref. ^23^), to compare with top-down and sideways constraints. The NWE framework will help them do that by promoting an understanding of the underlying processes and providing the data sets and calculations needed to parameterize and benchmark the next generation of TBMs built from the perspective of the atmosphere-land-water continuum of carbon flows.

## NWE across landscapes and biomes

To date, most detailed carbon budget studies have been undertaken at fine spatial scales focusing on individual ecosystem types, including forest stands^90,91^, agricultural lands^38,92^, wetlands^93,94^, and individual lakes and streams^95,96^. However, little is known about the complete carbon budget of heterogeneous landscapes composed of a diverse mosaic of terrestrial and aquatic ecosystems^41^. Upscaling from individual ecosystems to heterogeneous landscapes and biome scales still represents a major challenge for researchers and a top priority for policymakers^36,86^. It is likely that the contribution of different ecosystem types to carbon accumulation and exchange with the atmosphere varies largely across scales, landscape types, and biomes. Yet there are surprisingly few studies that have attempted to integrate terrestrial and aquatic carbon fluxes and that can be used to test this premise. In the section below we describe three such studies that have carried out integrative carbon budget assessments for contrasting types of landscapes. Note that these studies did not follow the NWE framework as described here, and none of them provide direct measurements of terrestrial and aquatic carbon accumulation; in all cases carbon accumulation (or loss) for each ecosystem type were calculated using NECBs. We nevertheless use these studies as examples to highlight the interest and also the challenges of applying the NWE framework across landscape types.

### North temperate lake district (Fig. 2a)

**Fig. 2:**
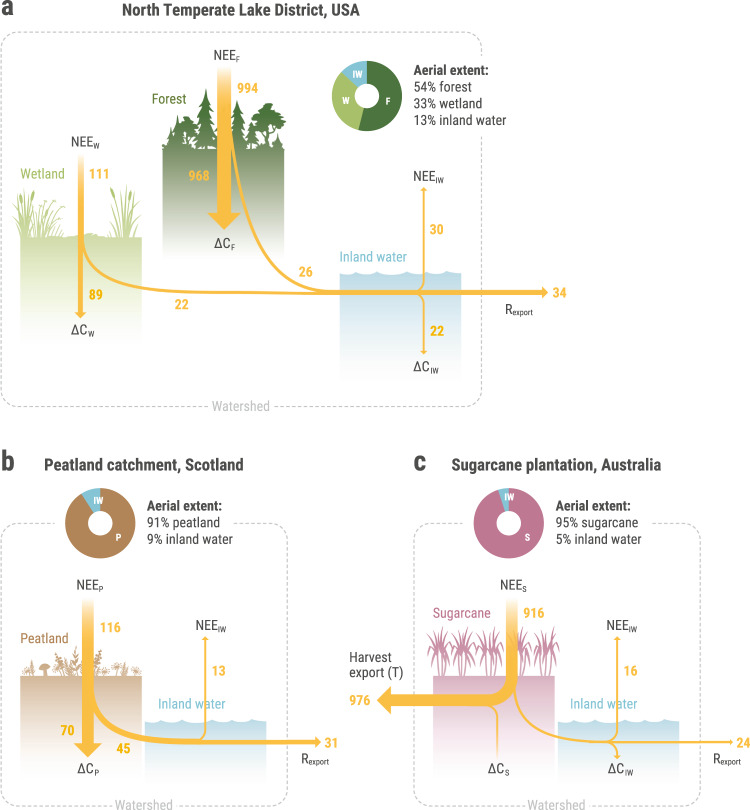
Examples of integrative carbon budgets across different landscape types. The arrows’ width shows the relative magnitude of carbon flux and is relative within, but not among each case study. **a** North Temperate Lakes LTER site^36^; **b** Ombrotrophic peatland catchment in southern Scotland^97^; **c** Sugarcane plantation in Australia^38^. *∆C*_*IW*_ in the peatland example (panel **b**) was not measured and assumed negligible. Units are Gg C yr^−1^ in panel **a**, and g C m^−2^ yr^−1^ in panels **b** and **c**. NEE: Net Ecosystem Exchange, *∆C*: ecosystem carbon accumulation (or loss).

The study of inland carbon biogeochemistry has been dominated by work in northern temperate and boreal ecosystems. The role of forests, wetlands, and lakes in the carbon budgets of these regions is relatively well understood. For example, a complete analysis of the terrestrial and aquatic lateral and vertical carbon fluxes was conducted for one north temperate region—the Northern Highlands Lake District in northern Wisconsin, USA^36^ (Fig. 2a). In this system, the lateral transfer of carbon from forests and peat-containing wetlands to lakes was substantial, and although wetlands and lakes make up a total of 33% of the landscape area, the sediments of these water bodies accounted for more than 80% of the total fixed carbon pool^36^. Given this large sedimentary pool of carbon, net carbon export out of the system through rivers was low.

### Peatland catchment (Fig. 2b)

Although peatlands cover only a small area of the earth’s surface (2–3%), they are responsible for storing over 30% of the world’s total soil carbon pool^97^. Peatlands differ from the example above by lacking a strict terrestrial component, and instead only contain peatland and streams (Fig. 2b). A thorough evaluation of the carbon budget over 2 years in Auchencorth Moss, Scotland, showed that ~12% of the peatland carbon uptake returns to the atmosphere through stream carbon evasion, and that another ~30% is hydrologically exported downstream (mostly as dissolved organic carbon; DOC). Hence, although the peatland represents a strong sink of atmospheric carbon, only 58% of the uptake actually accumulates (i.e., is sequestered) in the landscape over the longer-term (Fig. 2b, ref. ^97^).

### Sugarcane plantation (Fig. 2c)

Agroecosystems are predominantly an anthropogenic land use and will thus often involve various anthropogenic carbon fluxes (*T* term in Eq. 3). For example, anthropogenic carbon inputs to agroecosystems may include the addition of animal manure or plant-based compost to agricultural fields, fertilizers, soil conditioners (e.g., lime), and livestock feed^92^. Furthermore, in addition to aquatic carbon export, carbon is laterally removed from the watershed via food, wood, or fiber export. Because harvest removals often represent a large portion of terrestrial NEP, the contribution of some agroecosystems to the net watershed carbon sink can be small^98^. Animal grazing, residue burning after harvesting, and CO_2_ emissions from irrigation water sourced from deep groundwater may also contribute to NWE^99^.

The hydrologic boundaries of agricultural watersheds are more human defined than those in natural landscapes. For example, the canalization and pumping of water for irrigation may involve lateral aquatic carbon exchanges with adjacent watersheds that are not captured by the riverine export term. Thus, lateral transfer of carbon into and out of the watershed through artificial water routing needs to be explicitly accounted for.

Figure 2c represents an example of a carbon budget in an agroecosystem involving terrestrial and aquatic exchanges. Here, the carbon balance of a sugarcane plantation in Australia was monitored over one year, which included the NEE of the plantation as well as the emission (CO_2_ and CH_4_) and export (organic plus inorganic) of carbon from drainage channels^38^. During this study, terrestrial carbon uptake was large, at 900 g C m^−2^ yr^−1^, with aquatic lateral export representing only a small fraction of terrestrial NEE (~3%). Taking into account large export losses of biomass due to the cultivation of sugarcane, carbon accumulation was close to neutral during that year.

## The way forward

Estimating land-atmosphere carbon exchange through the NWE framework will require accurate estimates of carbon accumulation in terrestrial and aquatic ecosystems as well as riverine export through the watershed outlet. In this section, we provide an overview of the current methodologies to measure these components, including how to quantify them at the watershed scale, and further discuss their current level of understanding and data availability (Table 1).

**Table 1:** Elements involved in the calculation of net watershed exchange (NWE), approaches to measure them, and current level of understanding

Term	Symbol	How to measure	Level of understanding
Forest carbon accumulation	∆C_F_	Inventories, process-based models, NECB. Important to consider hydrologic carbon transfers in process-based models	Medium to high. Carbon inventory data available in many regions due to forestry, but difficulty in quantifying contemporary soil carbon accumulation. Only a few TBMs account for the lateral hydrologic transfer of carbon
Wetland carbon accumulation	∆C_W_	Inventories, process-based models, NECB. Important to consider hydrologic carbon transfers in process-based models	Low. Very high heterogeneity and diversity of wetland features, no standardized way of assessing carbon accumulation and high uncertainty of lateral hydrologic transfer estimates in process-based models
Agroecosystems carbon accumulation	∆C_A_	Inventories, NECB, soil organic carbon changes. Important to include aquatic systems	Medium. Well studied for specific crops and land uses, but often highly heterogeneous at landscape scale. Agricultural aquatic areas seldom included in carbon accounting
Inland water carbon accumulation	∆C_IW_	^210^Pb-dating coupled to carbon content, carbon burial model	Medium. Substantial uncertainty related to lake sediment focusing and the role of vegetated littoral zones. Data paucity for most climate regions. Large river floodplains could play a substantial role (e.g., in the lower Amazon)
Riverine carbon export from the watershed	R_Export_	Discharge and carbon concentration measurements. It must include dissolved and particulate organic carbon (DOC and POC) as well as dissolved inorganic carbon (DIC)	High. Standardized and well-established measurement technique. Extreme discharge events (e.g., storm or snow melt events) are highly important and must be accounted for
Lateral transfer of product carbon in trade (exports minus imports)	T	Extract crop and wood products trade volume from publicly available statistical economic data, and convert crop biomass/wood volume to carbon weight	Medium to high. Trade volumes are usually well documented, and there are standard carbon conversion factors for both crop and wood products

Depending on the landscape, additional ecosystem types should be considered.

*NECB* Net ecosystem carbon balance.

### Carbon accumulation in forests

Forest carbon accumulation is inventoried in the four pools defined by the Good Practice Guidance of the Intergovernmental Panel on Climate Change (IPCC): live biomass, dead wood, litter, and soil organic matter. Live biomass is a dynamic component that can usually be estimated with a good level of accuracy thanks to forest inventory programs^100,101^ and satellite images^102,103^. Nevertheless, uncertainties remain in predicting the long-term trajectory in biomass accumulation due to disturbance or changes in the drivers of forest growth and tree mortality due to global change^104,105^. Process-based models can also be used to estimate carbon accumulation in forests as long as they account for lateral hydrologic carbon transfers to inland waters.

Estimating the changes over time for the three other detrital pools can be more challenging, especially for soil carbon pools that represent the largest terrestrial carbon reservoir^106^. Variability at the sampling unit scale is typically very high and therefore involves an extensive sampling effort to detect changes^107,108^. Furthermore, sampling techniques are prone to bias due to changes in soil density and measurements usually conducted for a fixed depth^109^. Finally, the mechanisms responsible for accumulation or stabilization of the soil carbon pool have not been explicitly integrated into soil biogeochemistry modeling (ref. ^110^, but see ref. ^111^). Traditional models of soil carbon cycling are based on the recalcitrance of plant material, while new understanding suggests that organic matter physical and chemical protection against microbial degradation is of high importance^112–115^. In summary, changes in soil carbon pools remain difficult to assess and model^116^, and therefore projections are prone to large uncertainties. It is worth noting that recent international initiatives such as 4 per mille will potentially lead to systematic measurements of soil carbon sequestration rates across regions^117,118^, providing invaluable data that researchers could use within the NWE framework in the near future.

### Carbon accumulation in wetlands

Typically at the interface of terrestrial and aquatic environments, and exhibiting features consistent with both terrestrial and aquatic environments, wetlands have an enormous carbon accumulation capacity^119^. Unlike forests, wetland inventories are not conducted as often or at large spatial scales, making it more difficult to pin down wetland carbon accumulation as stock changes. One alternative method for determining wetland carbon accumulation is by using radionuclide tracers to model sedimentation rates. However, these models are based on key assumptions that can be easily violated by episodic events responsible for deposition occurring at the landscape scale^120^. Assumptions include near-constant sedimentation rates and steady-state equilibrium between soils and atmospheric supplies of ^210^Pb (refs. ^120–122^). Modeling efforts used to estimate timing of such events based upon ^210^Pb (ref. ^123^) exist and application of multiple tracers including ^137^Cs and ^14^C can be included to verify rates and obtain chronological markers in wetland ecosystems (e.g., refs. ^124,125^). However, carbon dating methods are inaccurate for near surface peat, making it challenging to determine recent accumulation rates responding to changes in climate or disturbance regimes^126^.

Another method to assess carbon accumulation in wetlands is to use process-based models. However, wetland models do not often account for lateral carbon transfers to streams and rivers (e.g., the McGill Wetland Model^127^, or CaMP^128^), leading to potentially biased estimates of wetland NEE and carbon accumulation. Wetland models tend to omit these transfers due to the complexity and number of parameters required to model the hydrological and biogeochemical dynamics needed to predict water flow rates as well as sorption/desorption processes of organic carbon to minerals. Simple scalable model relationships are required before carbon hydrological exports can be widely incorporated into wetland models, especially for large-scale models (see section “Challenges and opportunities” in the Supplementary Information).

Assessing carbon accumulation in wetlands at large spatial scales can be particularly challenging because of the large variation among wetland types with varying combinations of hydrogeomorphology, vegetation, and climate. For instance, depression wetlands with no surface channel connection to aquatic systems have been shown to accumulate significantly more carbon than riverine wetlands^129^. Similarly, in the Canadian boreal region, bog wetland types that are ombrotrophic and are dominated by Sphagnum mosses, tend to have larger sink capacity than fen wetlands that are connected to groundwater and are rich in sedge grasses^130^. Thus, a detailed mapping and classification of the different wetland types within the study watershed is central to accurately quantify the total wetland carbon accumulation.

### Carbon accumulation in agroecosystems

Food production occupies ~40% of earth’s land surface^131^ and understanding the role of agroecosystems in carbon accounting is thus essential. In terms of terrestrial carbon uptake, agroecosystems are often highly productive ecosystems compared to natural ecosystems due to intensified land, nutrient, and water management^41,98^. Croplands and horticulture can be characterized by periods of high atmospheric CO_2_ uptake during the growing seasons^132^, yet fallow fields with exposed soils can mineralize organic carbon at rates that may offset growing season carbon gains^133^. In grazing lands, the extent of the soil carbon sequestration capacity is driven by grass growth and manure inputs of recycled organic matter, which can be variable depending on nutrient availability and livestock density. Soil carbon accumulation in grasslands is likely to persist for longer timescales, which may prove significant on the watershed scale^134^.

Due to the diversity of land use and land management practices, agroecosystems require unique carbon accounting relative to natural ecosystems. Net carbon changes in agroecosystems are typically constrained by eddy covariance towers or inventories of soil organic carbon stock change^117,132^. However, there exist limitations in accurately representing landscape heterogeneity and quantifying long term changes, which are complicated by changes in crops, mechanical movement of soil, as well as the complex import and export of organic matter. Detecting soil carbon changes in agricultural fields requires sampling that represents all landscape components (i.e., flat, sloping, and depression areas) and accounts for the full plough depth^135^.

Artificial water bodies feature widely in agricultural landscapes and include ditches, irrigation canals, aquaculture ponds, and farm dams. The creation of artificial waters and how these fit into regional/global carbon budgets, which is largely unknown, highlights another reason why an integrated terrestrial-aquatic approach is needed moving forward. Aquatic areas on farms often have wetland-like characteristics that can promote carbon accumulation via intense aquatic primary production^136^. Furthermore, previous research demonstrates that both on-farm channels and dams can accumulate sediment by intercepting eroded soils and runoff with high sediment loads^137–139^. Thus, future measurements of sediment carbon accumulation in on-farm waterbodies and channel weirs will improve agroecosystem carbon accumulation estimates, as these waters capture a significant fraction of eroded soil that would otherwise be assumed lost^140,141^.

### Carbon accumulation in inland waters

Since the first attempt to integrate inland waters into the terrestrial carbon budget^142^, the estimates of global inland water carbon emissions have steadily increased as the result of a remarkable increase in aquatic carbon flux measurements worldwide and improvements in upscaling approaches^14^. In contrast, the estimates of global carbon accumulation in aquatic systems—mainly occurring in lakes and reservoirs—have barely been revised over the past 2 decades^14^, with the numbers oscillating between 0.15^142,143^ and 0.6 Pg C per year^144,145^. This in part reflects the strong emphasis placed on aquatic carbon emissions over the decades and a much smaller effort devoted to storage in aquatic sediments. Note, for example, that the extant data base on lake carbon accumulation remains modest and geographically biased, with only very few or no reports for more than 70% of the world’s regions^54,143^. Current research indicates that lake carbon accumulation has globally increased since industrial times^53,54^, with an acceleration in the recent decades that suggests dynamic scenarios where the lake carbon sink may be shifting upwards within relatively short time scales. Furthermore, reservoirs and impoundments have emerged as sites of intense carbon accumulation^143^, and this trend will accelerate in the future as reservoir construction keeps expanding globally^146^. The NWE framework brings aquatic carbon storage back to the forefront of limnological research as a centerpiece of the landscape carbon budget. Therefore, whereas quantifying aquatic carbon emissions remains central to identifying and constraining aquatic carbon sources and sinks, we call for a renewed interest in the quantification of lake and reservoir carbon accumulation as well as its projection under future climate and global change scenarios.

The current primary method to assess carbon accumulation in lakes and reservoirs is ^210^Pb-dating coupled to quantification of carbon content of sediment cores^54,147,148^. Alternatively, sediment accumulation rates can be calculated either directly by comparing a series of sequential, repeated bathymetric surveys performed in different years^138^ or using changes in storage capacity as a proxy to calculate volumetric sedimentation rates in reservoirs^149^. Newer approaches include sub-bottom sonar or laser-based technologies that allow detailed reconstruction of sediment basins within lakes and subsequent calculation of accumulation rates^150^. Current uncertainties in lake carbon storage arise from (1) variable estimates of the number and size distribution of lakes across regions and the globe^12,151–154^, (2) different lake basin shapes, (3) sediment heterogeneity and focusing, which may lead to bias when extrapolating site-specific burial rates to the whole lake basin^155,156^, (4) overlooking the role of macrophyte-rich, lake littoral areas with potentially high carbon accumulation rates, and (5) understudied processes that could increase the storage of carbon in sediments (e.g., calcite precipitation in high alkalinity lakes and reservoirs; ref. ^157^). Beyond overcoming these technical challenges, there is an acute need to improve the upscaling of carbon burial in lakes and reservoirs to the ensemble of systems at the regional and continental scales based on system, watershed and climate properties.

Besides lakes and reservoirs, future assessments of carbon accumulation in inland waters should consider other aquatic systems such as agricultural and urban ponds^139^ as well as large river corridors and their floodplains^158,159^ that may also contribute to the total carbon accumulation in the watershed. Carbon can also accumulate in small streams (e.g., leaves and branches that fall directly into the channel or the development of stream biofilms), especially in intermittent streams that recurrently run dry. Nonetheless, if the NWE mass balance (Eq. 3) is performed on a decadal or longer time basis, the carbon accumulated in small streams will likely have been remobilized during periodic flow events and either stored in lake and reservoir sediments or flushed out of the watershed.

### Riverine export

The riverine carbon export out of the watershed (*R*_*Export*_ in Eq. 3) is paramount for closing the watershed mass balance and is therefore of concern for each ecosystem-specific discipline. There are many decades of study of stream and river carbon export^160–162^, and there is both abundant literature and state-of-the-art methodology to facilitate the inclusion of this component. Hence, the level of understanding/accuracy of the riverine export term is high, relative to other elements of the watershed budget. River carbon export can be calculated as the product of river discharge and the concentration of the different organic and inorganic carbon forms, including DOC, particulate organic carbon, and dissolved inorganic carbon. For our purposes, particulate inorganic carbon is excluded because it generally derives from geological sources and is therefore disconnected from the contemporary atmospheric carbon flux. Previous research has shown that a large fraction of the annual riverine carbon export occurs during episodic, extreme flow events (snowmelt, storm, and hurricane events; refs. ^163,164^). For instance, Raymond and Saiers^165^ demonstrated that 86% of the annual export of DOC from temperate forests occurs during short but extreme flow events. Similarly, up to 60% of the annual export of particulate organic carbon may occur during storm events in tropical watersheds^166^. Hence, special efforts should be targeted at capturing these extreme discharge events. Automated, high-frequency measurements of discharge combined with discharge-concentration relationships have proven useful to obtain accurate estimates of annual riverine carbon export (e.g., ref. ^167^).

### Lateral transfer of product carbon in trade

The current best-practice recommendation is to separate the net trade carbon flux into gross fluxes of imports and exports, as well as separate reporting for crop products and wood products^8,48,168^. For example, the volumes of crop and wood products in international trade are available from the Food and Agriculture Organization of the United Nations^169^. Subsequently, the crop biomass is converted into dry matter and then into carbon using crop-specific factors^168,170^, and the wood products in volumetric units are converted to carbon weight using a mean wood density of 500 kg m^−3^, with a conversion factor of 0.45 as the carbon fraction in dry biomass^48^. The import and export of carbon in crop and wood products is typically tracked as it is moved across international borders and accounted for in the consumption or production reports of the nations involved^171^. Adapting this method to the NWE calculation, then, requires tracking of product trade across watershed boundaries: carbon is taken up from the atmosphere within the basin that the crop or wood is produced (i.e., grown and harvested) and subsequently stored in or released from the basin where it is processed and/or consumed. The study by Hayes et al.^172^. demonstrates the application of this method at sub-national scales by accounting for harvested products in the reporting zones (e.g., states and provinces in the U.S. and Canada) where they are produced, and then redistributing the carbon therein to the zones of consumption based on livestock and human populations and associated emissions factors. Resolving crop and wood product production and consumption within watersheds is more challenging where these data are not typically reported, but carbon transfers can be downscaled or aggregated to each basin using the best available data weighted by the area of producing sector (i.e., forestland and cropland) and per capita by livestock and human populations in the consuming sector.

### Scaling carbon accumulation to the watershed

Terrestrial carbon accumulation (or loss) from inventories and tower-based assessments can be interpolated or scaled up to the watershed unit using forest type and land cover maps and other spatial data from remote sensing. Meanwhile, process-based models can produce NWE-ready data by aggregating gridded model outputs to report them at watershed scales. Upscaling aquatic carbon accumulation to the watershed will be a product of specific carbon burial rates and area coverage of different inland water types (e.g., ref. ^149^), which will generally involve medium- to advanced-level GIS analyses. The quantification of aquatic carbon accumulation should preferably include the range of system types (rivers, lakes, reservoirs) of various characteristics (e.g., size, morphometry, chemistry) representative for the watershed of interest. Depending on the scale and variability of the watershed spatial unit, this may also include variability in burial rates associated with vegetation, climate, and land use. In upscaling terrestrial and aquatic carbon accumulation from point- and plot-based measurements, uncertainty assessments based on error propagation methods are highly recommended (e.g., Monte Carlo simulations).

### Reconciling spatial scales across methods

The development of regional carbon budgets requires an analysis framework to reconcile land-atmosphere carbon exchange from different estimation approaches at comparable spatial and temporal scales. This has been done in multi-year (often decadal scale) studies and mostly over broad geographies, such as biomes^81^ and continents^82^. In these studies, a common scale is used for reporting estimates within comparable spatial units. This is often determined by the most coarsely resolved data set in the comparison, and all of the other data sets are aggregated up to that scale^172^. Since land-atmosphere carbon exchange is what the atmosphere “sees” as one integrated flux, “bottom-up” inventory and modeled biospheric estimates are often compared at the scale of the “top-down” atmospheric constraint in these analyses^85^.

The emergence of NWE as a “sideways” constraint to the landscape carbon budget calls for a further unification of spatial scales across methods. Whereas atmospheric inversions and TBMs are flexible in terms of spatial unit delineation, NWE requires by concept a hydrologically defined landscape. Therefore, we can start with this sideways constraint to define the common analysis unit, i.e., the whole watershed above the spatial location of the *R*_*Export*_ measurement. Then, carbon budget assessments from inventories, in situ data, remote sensing, and TBMs could be aggregated by or scaled to watershed boundaries. Similarly, through the continued advancement of AIMs and approaches that use probabilistic error propagation techniques, we can spatially attribute atmospheric fluxes to whole watersheds. For carbon budget assessments that incorporate AIMs, the large watershed of higher-order river systems or drainage basins will likely be used for comparisons at coarse spatial scales. Alternatively, the same comparisons could be carried out for the smaller watersheds, but more observations and measurements will be required to constrain the budget in addition to the *R*_*Export*_ estimate.

If future carbon budget assessments use comparative hydrological units as suggested here, researchers will have at their disposal two independent reference fluxes, one top-down (atmospheric) and one sideways (riverine), that they can use in combination to constrain TBMs and inventory methods. This way, these two separate technical approaches will now be consistent in the assessment across the atmosphere-land-water continuum.

## Future challenges and opportunities

As with any other conceptual framework, there are challenges related to the deployment of NWE, which can and should be addressed in the future. As much as these aspects can be seen as challenges, they can also be turned into opportunities to pursue areas of watershed research that are in urgent need of attention and development. These challenges include, among others, identifying and quantifying potential groundwater-mediated carbon fluxes between adjacent watersheds and to the coastal ocean (see Supplementary Information for detailed discussion); incorporating features that are seldom included in landscape carbon budgets, such as urban and peri-urban areas, in a manner that is coherent with other landscape components; developing and sharing protocols to address the various components of the NWE equation in a consistent and comparable manner; and developing strategies to render the outcomes of NWE useful for management and mitigation strategies.

There is increasing consensus on the need to develop integrative landscape perspectives to address complex environmental issues^173–177^, in particular that consider the land to ocean aquatic continuum^46,178^, yet few studies actually do. The NWE framework offers a basis to link land and water processes within a watershed perspective, and although individual elements that compose the NWE equation may be routinely quantified and researched in many different landscapes across the world, there are actually very few examples of studies that integrate these elements within a single focus to derive a coherent landscape carbon budget. This is in part a reflection of the compartmentalized nature of carbon cycling studies, and also of the narrow vision of the funding and policies that underlie this research. The deployment of NWE or a similarly integrative framework requires not only a proactive stance from the research community to increase interaction and coordination between disciplinary groups, but also a fundamental shift in funding and research policy focus that will enable these interactions and explicitly support integrative studies.

The underlying premise of the NWE framework is that pursuing the current status quo of focusing on individual ecosystems in isolation from the other landscape elements will not fulfill the urgent need to map, quantify, understand, and eventually manage the continental sources and sinks of carbon. The NWE framework represents both a practical research tool that can be put to use towards that latter goal, and also a concrete platform to enable interaction and synergy between the different terrestrial, aquatic, and atmospheric research communities. It can serve as a basis to identify and address other challenges and opportunities, define common research goals and needs, and overcome technical, conceptual and even semantic obstacles that transcend disciplines. This communication and interaction are needed now more than ever in view of the urgency in improving current estimates of the continental carbon budget, and the fact that the development of climate mitigation strategies is an intrinsically interdisciplinary challenge.

## Supplementary information

Supplementary Information
