# Supplementary material for: Integrating terrestrial and aquatic ecosystems to constrain estimates of land-atmosphere carbon exchange
Source: Nat Commun. 2023 Mar 21;14:1571. doi: 10.1038/s41467-023-37232-2 (PMC10030657; doi:10.1038/s41467-023-37232-2)
Supplement: Supplementary file 1 — Supplementary Information [file 41467_2023_37232_MOESM1_ESM.pdf]

Supplementary Information

Integrating Terrestrial and Aquatic Ecosystems to Constrain Estimates of Land-Atmosphere Carbon Exchange

Joan P. Casas-Ruiz[1*†], Pascal Bodmer[2†], Kelly Ann Bona[3], David Butman[4], Mathilde Couturier[2], Erik J. S. Emilson[5], Kerri Finlay[6], Hélène Genet[7], Daniel Hayes[8], Jan Karlsson[9], David Paré[10], Changhui Peng[2], Rob Striegl[11], Jackie Webb[12], Xinyuan Wei[8], Susan E. Ziegler[13], Paul A. del Giorgio[2]

[1] Research Group on Ecology of Inland Waters, Institute of Aquatic Ecology, University of Girona, Girona, Spain
[2] Groupe de Recherche Interuniversitaire en Limnologie (GRIL), Département des sciences biologiques, Université du Québec à Montréal, Montréal, Québec, Canada
[3] Environment and Climate Change Canada, Gatineau, Quebec, Canada
[4] Department of Civil and Environmental Engineering, University of Washington, WA, USA
[5] Natural Resources Canada, Sault Ste. Marie, Ontario, Canada
[6] University of Regina, Regina, Saskatchewan, Canada
[7] University of Alaska Fairbanks, Fairbanks, Alaska, USA
[8] University of Maine, Orono, Maine, USA
[9] Umeå Universitet, Umeå, Sweden
[10] Natural Resources Canada, Québec, Québec, Canada
[11] United States Geological Survey, Boulder, Colorado, USA
[12] Centre for Regional and Rural Futures (CeRRF) Faculty of Science, Engineering and Built Environment Deakin University, New South Wales, Australia
[13] Memorial University of Newfoundland, St. John's, Newfoundland and Labrador, Canada

*Corresponding author: jpcasasruiz@gmail.com
†These authors contributed equally to this work

Accounting for lithospheric carbon inputs to inland waters

In its simplest form, eq. 3 does not capture the present-day inputs of ancient carbon from the lithosphere. In contrast, the storage in sediments and export through rivers of this same ancient carbon are included in the ΔC and R_{Export} terms. Hence, not accounting separately for lithospheric carbon fluxes may lead to biased NWE estimates. We can look at the case of petrogenic organic carbon (OC_{Petro}) as an example. On the one hand, if measurements of aquatic carbon storage (ΔC_{IW}) and export (R_{Export}) include the OC_{Petro} fraction, inputs of carbon from the lithosphere will be mistakenly allocated to contemporary watershed NPP, leading to NWE overestimation. On the other hand, aquatic emissions derived from OC_{Petro} remineralization are not captured in eq. 3 as is, which will therefore lead to aquatic NEE underestimation and, again, NWE overestimation.

From a theoretical point of view, there are two options to address this issue. First, researchers could subtract the fraction of lithogenic carbon from the storage (ΔC_{IW}) and export (R_{Export}) terms, and then add the lithogenic fraction of aquatic emissions as an additional emission term to the equation. There are multiple methodological challenges associated with separating the old fraction from the emission, storage, and export fluxes, and in the best-case scenario it would come with three additional sources of uncertainty. Second, researchers could estimate old carbon inputs from the lithosphere and soils to inland waters and add them to eq. 3 as an additional input term. Doing so would fully close the mass balance, avoiding the potential overestimation of NWE, and has the additional benefit that it only adds one source of uncertainty. Quantifying old carbon inputs to inland waters is not exempt from methodological challenges, yet given the above considerations, we recommend choosing this second option if possible. In the subsections below, we provide potential solutions to quantify lithospheric carbon inputs to aquatic systems and outline regions and watershed types where these could be significant. If lithogenic carbon fluxes are not accounted for in the carbon mass balance, the potential overestimation of NWE should be carefully evaluated.

For a hypothetical watershed where fluxes of petrogenic- and carbonate-derived ancient carbon are relevant, the NWE equation (eq. 3) should be expanded as follows:

$$NWE + OC_{Petro} + W_{CO_3^{2-}} = \sum_{i=1}^{n} \Delta C_i + R_{Export} + T \qquad \text{(eq. S1)}$$

where, $OC_{\text{Petro}}$and $W_{CO_3^{2-}}$ are the inputs to inland waters of OC_{Petro} and ancient dissolved inorganic carbon from carbonate dissolution, respectively. Researchers should assess *a priori* the potential contribution of each of these ancient carbon sources to the study watershed and adapt eq. S1 to their case.

Petrogenic organic carbon

The erosion and oxidation of OC_{Petro} releases CO_2 from long-term geological storage to the atmosphere. This flux, which is sometimes referred to as "geo-respiration", has recently been identified as an important component of the geological carbon cycle[1], and its quantification has received particular attention over the last decade. Eroded OC_{Petro} is mobilized on hillslopes and colluvial deposits and transferred to the particulate load of streams and rivers, from where it can be mineralized and emitted to the atmosphere, stored in sediments, or exported downstream. Recently, riverine dissolved fluxes of rhenium have proven to be an integrative proxy to OC_{Petro} erosion rates[2,3]. Therefore, if measurements of dissolved rhenium are performed at the outlet of the study watershed,

they could be used to quantify the entrance of eroded OC_{Petro} to streams and rivers at the watershed scale (OC_{Petro}in eq. S1). Alternatively, measurements of soil and bedrock OC coupled to ^{14}C activity measurements may provide estimates of average OC_{Petro} erosion and potential oxidation[4], although quantification at watershed scales will bring additional challenges and uncertainty.

Ancient DIC from carbonate mineral dissolution

All dissolved inorganic carbon (DIC) derived from silicate weathering originates from CO_2 derived from contemporary soil organic matter decomposition and/or direct reaction with atmospheric CO_2. Thus, silicate rock weathering simply changes the form of DIC, from CO_2 to bicarbonate. This is an example of the weathering reaction for enstatite ($MgSiO_3$):

$$MgSiO_3 + 2CO_2 + 3H_2O \leftrightarrow Mg_2^+ + 2HCO_3^- + H_4SiO_4 \quad (1)$$

In contrast, during chemical weathering of carbonate rocks, 2 mols of DIC are released for every mol of CO_2. One mol derives from the transformation of soil respiratory CO_2 to bicarbonate (same as for silicate rocks), and an extra mol is released through the direct dissolution of carbonate minerals. Here is an example for calcite ($CaCO_3$):

$$CaCO_3 + CO_2 + H_2O \leftrightarrow Ca_2^+ + 2HCO_3^- \quad (2)$$

Whereas the mol derived from the transformation of soil respiratory CO_2 is biogenic and contemporary, the one derived from carbonate minerals dissolution is geogenic and can be millions of years old. Hence, to fully close the short-term mass balance in eq. 3, an additional lithospheric input term of ancient geogenic DIC has to be added.

There are studies that provide empirical models to calculate the generation of bicarbonate or alkalinity for different lithologies based on local runoff[5–7]. Thus, if runoff and the fraction of carbonate geology in the study watershed are known, these empirical models can be applied to estimate the total weathering flux of bicarbonate at the watershed scale (e.g., ref [8]). The input of DIC derived from carbonate minerals dissolution can be then computed as half of the total weathering flux of bicarbonate. The GLiM database[9] contains global lithology data at high resolution that can be used for this purpose. The contribution of ancient, weathered carbonates will increase with the relative proportion of carbonate to silicate lithologies in the watershed.

As mentioned above, an alternative yet less convenient approach would consist of 1) subtracting the geogenic fraction of the river DIC export term and 2) adding a new equation term of aquatic emissions linked to geogenic DIC inputs[10,11]. The geogenic fraction of both fluxes can be assessed by mixing models based on the isotopic signatures (δ^{13}C) of riverine DIC and potential biogenic and geogenic endmembers (soil respiration and carbonate dissolution).

Challenges and opportunities

Groundwater exchange between watersheds

The global volume of groundwater to a depth of 2 km is estimated at 4.2 x 10^6 km^3, of which approximately 6% is within 100 m of the ground surface[12]. Across North America, groundwater DOC concentration oscillates around ~8.8 mg C L^{-1} at < 10m depth decreasing to ~3.3 mg C L^{-1} at 90 – 100m, while groundwater DIC averages 30 – 43 mg C L^{-1} and does not vary substantially with depth[12]. Combining these carbon concentration profiles with estimates of groundwater volume and discharge, Downing and Striegl[12] calculated a potential global groundwater carbon content of 145 Pg and a groundwater carbon discharge to inland waters of ~0.68 Pg C yr^{-1}.

Subsurface water flows within a watershed generally originate from the same watershed domain[13]. Thus, although subsurface carbon fluxes are difficult to quantify, they are all indirectly accounted for in the NWE framework through the whole-watershed carbon mass balance (Supplementary Note; see below). On the contrary, deeper flows paths such as groundwater upwellings in karstic springs can potentially derive from outside the study watershed and would therefore have an impact on NWE calculations if ignored. For example, in a small Costa Rican rainforest, Genereux *et al.*[14] showed that overlooking inputs of carbon associated to deep groundwater regional flow paths leads to the wrong impression that the whole rainforest (including streams) acts as a carbon sink when in reality it was a substantial source of CO_2 to the atmosphere. Hence, external deep groundwater carbon exchanges should be considered an additional input or output term in the watershed mass balance of carbon. A major challenge remains the identification and quantification of only those deep carbon fluxes that originate from outside of the study watershed. Multiple isotopic techniques, such as measuring the stable isotopes of water with radiogenic isotopes, can offer a robust approach to constraining both the source and age of groundwater inputs[15–17].

Using larger watersheds would nonetheless reduce the importance of deep groundwater carbon exchanges relative to the other terms of the mass balance (NWE, ΔC, and R_{Export}). This is because lateral groundwater exchanges into and out of the watershed would mostly occur along the perimeter of the watershed, whereas carbon exchange with the atmosphere and carbon accumulation take place across the total area of the watershed. Hence, assuming the watershed shape is held constant, increasing the watershed size will increase the area to perimeter ratio and therefore the magnitude of NWE, ΔC, and R_{Export} will increase faster than potential groundwater exchanges with adjacent watersheds.

Direct groundwater discharge to the ocean

Hydraulic gradients determined by topography and subsurface porosity in coastal regions often result in direct groundwater discharge from the continents into the ocean, usually referred to as fresh submarine groundwater discharge (fresh SGD). Unlike saline SGD, a diffusive pathway of recirculated seawater that is ubiquitous along the world's coastlines, fresh SGD is a more local, point source of terrestrially derived water and nutrients to the coastal ocean. This lateral transfer flux represents an important connection between the coastal ocean and the land. Yet, fresh SGD has been overlooked due to the difficulties to investigate it and thus is not accounted for in global carbon budgets, even though it is widely recognized as an important contributor to the hydrological and biogeochemical budgets of coastal and freshwater systems[18].

Recent advances in spatially explicit groundwater models constrain global fresh SGD to 289–2400 km^3 per year (refs.[19–21]). This is 0.6 to 6.4% of global river discharge[22] and represents at least 5% of marine Li, Mg, Ca, Sr, and Ba budgets[23] and ~2 % of total freshwater input of carbon, nitrogen, silica and strontium to the ocean[21], depending on which fresh SGD amounts are used. Applying groundwater concentration versus carbon depth profiles from Downing and Striegl [12], this amounts to a global fresh SGD of 10–86 Tg C yr^{-1} and 2–17 Tg C yr^{-1} of dissolved inorganic carbon and DOC, respectively. Such a lateral transfer flux represents an additional hydrologic carbon export out of coastal watersheds that should be considered when applying the NWE framework to watersheds with a coastal component. This is especially true in regions with substantial fresh SGD relative to river discharge such as in tropical coastlines as well as high-relief, tectonically active margins[20].

Incorporating hydrologic carbon transfers into wetland models

Process-based models offer an opportunity to estimate carbon accumulation in wetlands, but only if the lateral transfer of carbon to inland waters is accounted for. As of today, the lateral carbon transfer from wetlands to streams and rivers remains challenging to quantify, predict and, therefore, to incorporate into the structure of process-based models. At the same time furthering the NWE approach offers some opportunity to bridge this gap and thereby improve wetland models.

Lateral export of carbon from wetlands is a function of the soil porewater concentrations of mobile carbon species and the hydrology responsible for mobilizing them. Water table changes have been linked to peatland CO_2 and CH_4 dynamics[24] and are typically used in process-based models [25]. Nevertheless, it remains unclear how hydrology impacts soil porewater DOC. Beyond simple dilution, changes in acidity and ionic strength associated with changes in water table levels, runoff, or drought conditions exerts a solubility control on DOC concentration in porewaters[26,27]. In some peatlands, for example, soil acidity is the major factor predicting porewater DOC[28], emphasizing that hydrologically driven chemical and biological processes are required in predicting lateral carbon fluxes in wetlands. Furthermore, lateral transfers of DOC may also be underestimated since they typically occur outside of the growing season, an underrepresented study period in higher latitude wetlands [29,30]. Thus, a predictive understanding of lateral carbon transfers is needed in order to incorporate them into wetland carbon budgets and process-based models, especially for DOC but also dissolved inorganic carbon.

Although increasing our understanding of chemical and biological processes that could control wetland DOC export will be highly valuable for improving process-based models, the scalability of these relationships will be just as important to consider. In order to integrate DOC export into national- or regional-scale models used for greenhouse gas inventories and mitigation plans, wetland researchers will need to consider parameters available at large spatial-scales. For instance, simple classes should be explored such as catchment-scale hydrological units (e.g., hydrological response units; ref. [31]) that could be used as an index of lateral flow strength. Also, peatland functional types that could reflect porewater pH as well as widely available climate variables that indicate drought conditions (e.g., the Drought Code of the Canadian Forest Fire Weather Index System; ref. [32]) should be considered.

Resolving lateral carbon transfers at relevant scales presents some challenges that could be turned into opportunities using the NWE approach. Many wetlands are “marginal” environments that exist at the interface between terrestrial and aquatic ecosystems and are thus difficult to resolve discretely both in terms of their landscape extent (e.g., forested wetlands) and contribution to lateral carbon transfers[33]. If lateral carbon transfers are directed into an adjacent hillslope, how are they

resolved and accounted for? If riparian wetlands contribute to aquatic carbon fluxes, how are they resolved and accounted for? Incorporating wetlands into the NWE approach would enable this accounting. The prediction for lateral carbon transport for identified wetlands within a watershed should contribute to the overall watershed carbon export and thus enable means to constrain such lateral transfers fluxes between wetlands and the terrestrial environment. Further, by observing export at the smaller watershed scale, concentration-discharge relationships can be used to identify the more cryptic riparian wetlands, as such near stream sources that exhibit strong clockwise hysteresis patterns with discharge[34,35].

Supplementary Note | Accounting for lateral hydrologic carbon exchanges

The net accumulation of carbon in an ecosystem does not necessarily reflect the amount of carbon it exchanges with the atmosphere. Terrestrial ecosystems often accumulate less carbon than they withdraw from the atmosphere[36], and even more evident, aquatic systems are substantial carbon sources to the atmosphere while simultaneously accumulating carbon in their sediments[37]. In the absence of lateral anthropogenic carbon fluxes, the reason behind the mismatch between carbon accumulation and exchange with the atmosphere is often the same for both cases, namely the hydrologic transfer of carbon from terrestrial to aquatic ecosystems. Therefore, whereas the mismatch can be substantial for terrestrial and aquatic ecosystems separately, they offset each other when both ecosystems are combined. This can be illustrated with a simple numerical exercise. The figure below shows a simplified scheme of the vertical and horizontal carbon fluxes across the terrestrial and aquatic ecosystems within a model watershed. We have assigned random numbers to each of these fluxes based on the principle of conserving the overall mass balance of the different components. To make the mass balance straightforward, we assume steady state conditions (i.e., fluxes are constant through time) and momentaneously define NEE as positive when there is a net flux from the atmosphere to the watershed (continued on next page).

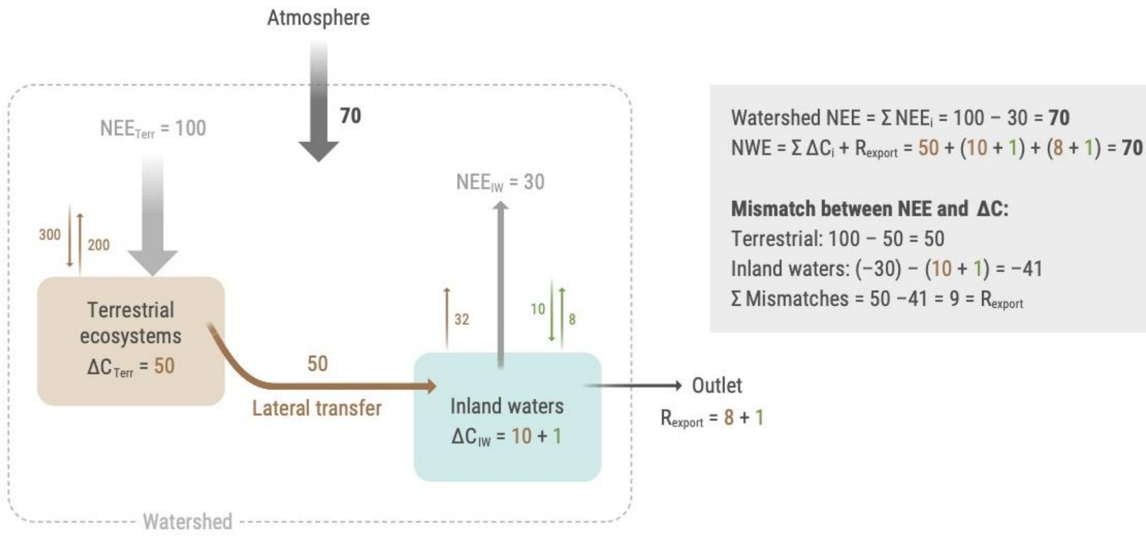

$$\text{Watershed NEE} = \Sigma\,\text{NEE}_i = 100 - 30 = \mathbf{70}$$
$$\text{NWE} = \Sigma\,\Delta C_i + R_{export} = 50 + (10 + 1) + (8 + 1) = \mathbf{70}$$

Mismatch between NEE and ΔC:
Terrestrial: 100 − 50 = 50
Inland waters: (−30) − (10 + 1) = −41
Σ Mismatches = 50 −41 = 9 = R_{export}

Simplified scheme of the different ecosystems and carbon fluxes across a theoretical watershed. All fluxes are in absolute values, with the shape of the arrow indicating their direction. Brown and green elements correspond to carbon originally fixed from the atmosphere by terrestrial and aquatic ecosystems, respectively. Note that, against convention, positive NEE indicates an input from the atmosphere to the landscape, while negative NEE indicates emission to the atmosphere.

According to this simulated scheme, the total gross primary production and ecosystem respiration in terrestrial ecosystems are 300 and 200 carbon units of mass per time (C t^{-1}; e.g., tons of carbon per year), respectively. Thus, the net carbon exchange between terrestrial ecosystems and the atmosphere (NEE_{Terr}) is 100 C t^{-1}, i.e., net uptake. Fifty units out of the 100 are stored *in situ* (ΔC_{Terr}), for example as forest and wetland biomass, while the other half is laterally transferred as organic and inorganic carbon to inland waters (*Lateral Transfer*). This induces a mismatch between NEE_{Terr} and ΔC_{Terr} of 50 C t^{-1}, implying we would underestimate NEE_{Terr} if assumed equivalent to ΔC_{Terr}. Inland waters then receive this terrestrial *Lateral Transfer*, that is, 50 C t^{-1} of terrestrial organic and inorganic carbon. Thirty-two units out of the 50 are emitted from streams, rivers, and lakes to the atmosphere (NEE_{IW}), either through the direct emission of terrestrial inorganic carbon (i.e., soil-respiratory CO_2 and CH_4) or through the mineralization of terrestrial organic carbon within aquatic systems. In addition, 10 C t^{-1} of the terrestrial *Lateral Transfer* are buried at the bottom of lakes and stored in the sediment pool (ΔC_{IW}). Simultaneously, aquatic ecosystems fix carbon directly from the atmosphere (10 C t^{-1}). Most of this atmospheric carbon fixed by aquatic ecosystems is readily remineralized and outgassed (8 C t^{-1}), whereas some of it is stored in lake sediments (1 C t^{-1}). Together, this represents a mismatch between NEE_{IW} and ΔC_{IW} of -41 C t^{-1}, implying that, if only based on carbon accumulation, we would assume a net uptake of 11 C t^{-1} from the atmosphere when aquatic systems are in reality emitting 30 C t^{-1} to the atmosphere. The remaining 9 C t^{-1} of the terrestrial lateral transfer plus carbon fixed by aquatic ecosystems are transported as DOC, POC, and DIC by rivers to the outlet of the watershed (R_{Export}).

From an individual ecosystem perspective, it becomes clear that ΔC does not represent NEE, because there is a clear mismatch both in terrestrial (50 C t^{-1}) and aquatic ecosystems (-41 C t^{-1}). Nevertheless, we have a mismatch in terrestrial ecosystems because part of the carbon uptake is laterally transferred to inland waters, and we have a mismatch in inland waters because a large share of the aquatic fluxes is actually a relocation of the transferred terrestrial carbon. Hence, when we adopt a holistic perspective and combine both terrestrial and aquatic ecosystems through a mass balance, the two mismatches practically offset each other. In fact, the offset of mismatches is not absolute but equivalent to the watershed riverine export [50 + (-40) = 9 C t^{-1}], which makes sense if one considers that the export at the watershed outlet represents the imbalance between watershed-atmosphere carbon exchange and watershed carbon accumulation. Therefore, by combining the accumulation of carbon in the different terrestrial and aquatic elements and the riverine carbon export at the watershed outlet, we indirectly account for all hydrologic lateral exchanges of carbon among ecosystems. This simple numerical exercise shows the power of adopting a whole-watershed perspective for landscape carbon accounting and emphasizes the central need to consider all the different ecosystem types together.

Supplementary references

1. Hilton, R. G. & West, A. J. Mountains, erosion and the carbon cycle. *Nat. Rev. Earth Environ.* **1**, 284–299 (2020).
2. Hilton, R. G., Gaillardet, J., Calmels, D. & Birck, J.-L. Geological respiration of a mountain belt revealed by the trace element rhenium. *Earth Planet. Sci. Lett.* **403**, 27–36 (2014).
3. Dalai, T. K., Singh, S. K., Trivedi, J. R. & Krishnaswami, S. Dissolved rhenium in the Yamuna river system and the Ganga in the Himalaya: role of black shale weathering on the budgets of Re, Os, and U in rivers and CO2 in the atmosphere. *Geochim. Cosmochim. Acta* **66**, 29–43 (2002).
4. Hemingway, J. D. *et al.* Microbial oxidation of lithospheric organic carbon in rapidly eroding tropical mountain soils. *Science (80-.).* **360**, 209–212 (2018).
5. Amiotte Suchet, P., Probst, J.-L. & Ludwig, W. Worldwide distribution of continental rock lithology: Implications for the atmospheric/soil CO 2 uptake by continental weathering and alkalinity river transport to the oceans. *Global Biogeochem. Cycles* **17**, (2003).
6. Hartmann, J., Jansen, N., Dürr, H. H., Kempe, S. & Köhler, P. Global CO2-consumption by chemical weathering: What is the contribution of highly active weathering regions? *Glob. Planet. Change* **69**, 185–194 (2009).
7. Jansen, N. Chemical rock weathering as source of dissolved silica and sink of atmospheric CO2. PhD Thesis. *University of Hamburg* (University of Hamburg, 2010).
8. Marcé, R. *et al.* Carbonate weathering as a driver of CO2 supersaturation in lakes. *Nat. Geosci.* **8**, 107–111 (2015).
9. Hartmann, J. & Moosdorf, N. The new global lithological map database GLiM: A representation of rock properties at the Earth surface. *Geochemistry, Geophys. Geosystems* **13**, 1–37 (2012).
10. Billett, M. F., Garnett, M. H. & Dinsmore, K. J. Should Aquatic CO2 Evasion be Included in Contemporary Carbon Budgets for Peatland Ecosystems? *Ecosystems* **18**, 471–480 (2015).
11. Duvert, C. *et al.* Groundwater-Derived DIC and Carbonate Buffering Enhance Fluvial CO2 Evasion in Two Australian Tropical Rivers. *J. Geophys. Res. Biogeosciences* **124**, 312–327 (2019).
12. Downing, J. A. & Striegl, R. G. Size, age, renewal, and discharge of groundwater carbon. *Inl. Waters* **2041**, 1–6 (2018).
13. Fan, Y. Are catchments leaky? *WIREs Water* **6**, 1–25 (2019).
14. Genereux, D. P., Nagy, L. A., Osburn, C. L. & Oberbauer, S. F. A connection to deep groundwater alters ecosystem carbon fluxes and budgets: Example from a Costa Rican rainforest. *Geophys. Res. Lett.* **40**, 2066–2070 (2013).
15. Moore, K. B., Ekwurzel, B., Esser, B. K., Hudson, G. B. & Moran, J. E. Sources of groundwater nitrate revealed using residence time and isotope methods. *Appl. Geochemistry* **21**, 1016–1029 (2006).
16. Maher, D. T. *et al.* Blue carbon oxidation revealed by radiogenic and stable isotopes in a mangrove system. *Geophys. Res. Lett.* **44**, 4889–4896 (2017).
17. Wang, Q. *et al.* Quantification of the water age and submarine groundwater discharge in a typical semi-enclosed bay using stable oxygen (18O) and radioactive radium (228Ra) isotopes. *J. Hydrol.* **603**, 127088 (2021).
18. Santos, I. R. *et al.* Submarine groundwater discharge impacts on coastal nutrient biogeochemistry. *Nat. Rev. Earth Environ.* (2021) doi:10.1038/s43017-021-00152-0.
19. Beck, A. J., Charette, M. A., Cochran, J. K., Gonneea, M. E. & Peucker-Ehrenbrink, B. Dissolved strontium in the subterranean estuary - Implications for the marine strontium isotope budget. *Geochim. Cosmochim. Acta* **117**, 33–52 (2013).
20. Zhou, Y. Q., Sawyer, A. H., David, C. H. & Famiglietti, J. S. Fresh Submarine Groundwater Discharge to the Near-Global Coast. *Geophys. Res. Lett.* **46**, 5855–5863 (2019).
21. Luijendijk, E., Gleeson, T. & Moosdorf, N. Fresh groundwater discharge insignificant for the

world's oceans but important for coastal ecosystems. *Nat. Commun.* **11**, (2020).
22. Dai, A. & Trenberth, K. E. Estimates of Freshwater Discharge from Continents: Latitudinal and Seasonal Variations. *J. Hydrometeorol.* **3**, 660–687 (2002).
23. Mayfield, K. K. *et al.* Groundwater discharge impacts marine isotope budgets of Li, Mg, Ca, Sr, and Ba. *Nat. Commun.* **12**, 148 (2021).
24. Blodau, C. & Moore, T. R. Experimental response of peatland carbon dynamics to a water table fluctuation. *Aquat. Sci.* **65**, 47–62 (2003).
25. Bona, K. A. *et al.* The Canadian model for peatlands (CaMP): A peatland carbon model for national greenhouse gas reporting. *Ecol. Modell.* **431**, 109164 (2020).
26. Clark, J. M., Chapman, P. J., Adamson, J. K. & Lane, S. N. Influence of drought-induced acidification on the mobility of dissolved organic carbon in peat soils. *Glob. Chang. Biol.* **11**, 791–809 (2005).
27. Clark, J. M., Chapman, P. J., Heathwaite, A. L. & Adamson, J. K. Suppression of Dissolved Organic Carbon by Sulfate Induced Acidification during Simulated Droughts. *Environ. Sci. Technol.* **40**, 1776–1783 (2006).
28. Clark, J. M., Heinemeyer, A., Martin, P. & Bottrell, S. H. Processes controlling DOC in pore water during simulated drought cycles in six different UK peats. *Biogeochemistry* **109**, 253–270 (2012).
29. Waddington, J. M. & Roulet, N. T. Carbon balance of a boreal patterned peatland. *Glob. Chang. Biol.* **6**, 87–97 (2000).
30. Repo, E. *et al.* Release of CO2 and CH4 from small wetland lakes in western Siberia. *Tellus B Chem. Phys. Meteorol.* **59**, 788–796 (2007).
31. Devito, K. *et al.* A framework for broad-scale classification of hydrologic response units on the Boreal Plain: is topography the last thing to consider? *Hydrol. Process.* **19**, 1705–1714 (2005).
32. Van Wagner, C. E. & Forest, P. Development and structure of the canadian forest fireweather index system. in *Can. For. Serv., Forestry Tech. Rep* (Citeseer, 1987).
33. Creed, I. F., Sanford, S. E., Beall, F. D., Molot, L. A. & Dillon, P. J. Cryptic wetlands: integrating hidden wetlands in regression models of the export of dissolved organic carbon from forested landscapes. *Hydrol. Process.* **17**, 3629–3648 (2003).
34. Ågren, A. *et al.* Dissolved organic carbon characteristics in boreal streams in a forest-wetland gradient during the transition between winter and summer. *J. Geophys. Res. Biogeosciences* **113**, (2008).
35. Strohmeier, S. *et al.* Concentrations and fluxes of dissolved organic carbon in runoff from a forested catchment: insights from high frequency measurements. *Biogeosciences* **10**, 905–916 (2013).
36. Hayes, D. J. *et al.* Chapter 2: The North American Carbon Budget. Second State of the Carbon Cycle Report. *Second State Carbon Cycle Rep. A Sustain. Assess. Rep.* 71–108 (2018) doi:10.7930/SOCCR2.2018.Ch2.
37. Tranvik, L. J. *et al.* Lakes and reservoirs as regulators of carbon cycling and climate. *Limnol. Oceanogr.* **54**, 2298–2314 (2009).
